# Use of Fluorochrome-Labeled Inhibitors of Caspases to Detect Neuronal Apoptosis in the Whole-Mounted Lamprey Brain after Spinal Cord Injury

**DOI:** 10.1155/2012/835731

**Published:** 2012-07-08

**Authors:** Antón Barreiro-Iglesias, Michael I. Shifman

**Affiliations:** Shriners Hospitals Pediatric Research Center (Center for Neural Repair and Rehabilitation), Temple University School of Medicine, 3500 North Broad Street, Philadelphia, PA 19140, USA

## Abstract

Apoptosis is a major feature in neural development and important in traumatic diseases. The presence of active caspases is a widely accepted marker of apoptosis. We report here the development of a method to study neuronal apoptotic death in whole-mounted brain preparations using fluorochrome-labeled inhibitors of caspases (FLICA). As a model we used axotomy-induced retrograde neuronal death in the CNS of larval sea lampreys. Once inside the cell, the FLICA reagents bind covalently to active caspases causing apoptotic cells to fluoresce, whereas nonapoptotic cells remain unstained. The fluorescent probe, the poly caspase inhibitor FAM-VAD-FMK, was applied to whole-mounted brain preparations of larval sea lampreys 2 weeks after a complete spinal cord (SC) transection. Specific labeling occurred only in identifiable spinal-projecting neurons of the brainstem previously shown to undergo apoptotic neuronal death at later times after SC transection. These neurons also exhibited intense labeling 2 weeks after a complete SC transection when a specific caspase-8 inhibitor (FAM-LETD-FMK) served as the probe. In this study we show that FLICA reagents can be used to detect specific activated caspases in identified neurons of the whole-mounted lamprey brain. Our results suggest that axotomy may cause neuronal apoptosis by activation of the extrinsic apoptotic pathway.

## 1. Introduction

Studies in the basic neurosciences are heavily reliant upon rat and mouse models (for a review see [1]). Seventy-five percent of current research efforts are directed to rat mouse, and human brains, which represent 0.0001% of the nervous systems on the planet [1]. In recent decades, an increased number of studies have shown the usefulness of nonmammalian models for understanding developmental, pathological, and regenerative processes of the nervous system. Lampreys and fishes, for example, have proven to be valuable animal models for studying successful regeneration in the mature central nervous system (CNS) [2–5].

Lampreys occupy a key position close to the root of the vertebrate phylogenetic tree [6] and are thought to have existed largely unchanged for more than 500 million years, which makes them important animals from the standpoint of molecular evolution [7–10]. The unique evolutionary position of lampreys as early-evolved vertebrates, the sequencing of the sea lamprey (*Petromyzon marinus* L.) genome, and the adaptation and optimization of many established molecular biology and histochemistry techniques for use in this species have made it an emerging nonmammalian model organism of choice for investigations into the evolution of hallmark vertebrate characteristics. In addition, the sea lamprey has proven to be a valuable animal model in spinal cord (SC) injury and regeneration studies [2, 3].

Programmed cell death (apoptosis) of large numbers of immature neurons is a major feature in neural development. Neuronal cell death is also an important component of both acute and chronic neurodegenerative diseases and traumatic injuries. In apoptosis, caspases are responsible for proteolytic cleavages that lead to cell disassembly (effector caspases) and are involved in upstream regulatory events (initiator caspases) [11]. In vertebrates, caspase-dependent apoptosis occurs through two main pathways, the intrinsic and extrinsic pathways [12]. The intrinsic or mitochondrial pathway is activated by diverse stimuli, including genomic and metabolic stress, unfolded proteins, and other factors that lead to permeabilization of the outer membrane of the mitochondria and release of apoptotic proteins into the cytosol. Progression through the intrinsic pathway usually leads to activation of the initiator caspase-9 [11]. The extrinsic or death receptor pathway involves the activation of transmembrane death receptors by their ligands. This process usually leads to the activation of the initiator caspase-8 (or caspase-10; [11]). Therefore, activation of caspases serves as a hallmark of apoptosis [13].

Activated caspases can be detected in histological preparations and *in situ* in individual cells immunocytochemically with antibodies generated against the epitope that is characteristic of the cleaved, enzymatically active form of caspases [14, 15]. However, most commercially available antibodies have been generated against mammalian caspase protein sequences and therefore could be much less specific [16–18] when used in nonmammalian species. Thus the use of nonmammalian models for neuroscience research makes it necessary to develop new methods or adapt already existing methods to the specific characteristics of these animals, since most of the commercially available tools have been designed for use in mammals.

In this paper we propose to use the lamprey brain as a model system to develop a new method for detecting activated caspases in the whole-mounted CNS of nonmammalian species. Recent studies have shown that axotomy after a complete SC transection induces death of spinal-projecting neurons of the lamprey brainstem [19, 20]. The appearance of TUNEL staining in these neurons has suggested that they die by apoptosis [19].

**Figure 1:**
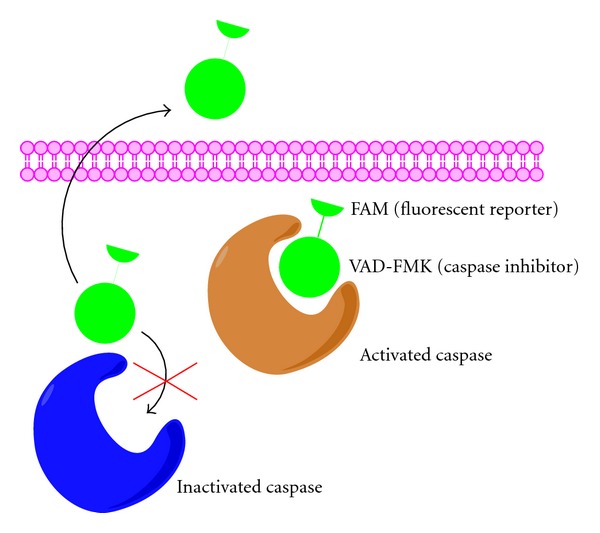
Schematic drawing illustrating how the FLICA method allows labeling of live cells that have activated caspases. Unbound FLICA reagent is washed out during the washing process (left side), whereas it attaches covalently to active caspases (right side).

We are reporting a new method for the detection of activated caspases based on the use of fluorochrome-labeled inhibitors of caspases (FLICA). When applied to live cells, cell-permeate FLICA reagents tagged with fluorescent molecules exclusively label cells that have active caspases and are undergoing apoptosis (see Figure 1; [13]). Because labeling requires that cells to be alive when the FLICA reagents are applied, they have only been used previously to detect activated caspases in cell culture assays [13]. This is the first study reporting the use of FLICA reagents to detect neuronal apoptosis and activated caspases in whole-mounted brain preparations of a vertebrate species *ex vivo*.

## 2. Experimental Procedures

### 2.1. Animals

Wild-type larval sea lampreys (*Petromyzon marinus* L.), 10–14 cm in length (4–7 years old), were obtained from streams feeding Lake Michigan and maintained in freshwater tanks at 16°C with the appropriate aeration until the day of use. Experiments were approved by the Institutional Animal Care and Use Committee at Temple University. Before experiments, animals (*n* = 25) were deeply anesthetized by immersion in Ringer solution containing 0.1% tricaine methanesulfonate (Sciencelab, Houston, TX, USA).

### 2.2. Spinal Cord (SC) Transection

The SC was exposed from the dorsal midline at the level of the fifth gill. Complete transection of the SC was performed with Castroviejo scissors, after which animals were kept on ice for 1 hour to allow the wound to air dry. The animals are kept during this hour on a paper towel soaked with ringer solution not in direct contact with the ice; the low temperature keeps the animals alive while allowing the wound to air dry. Each transected animal was examined 24 hours after surgery to confirm that there was no movement caudal to the lesion site. A transection was tentatively considered complete if the animal could move only its head and body rostral to the lesion. Animals were allowed to recover in aerated fresh water tanks at room temperature.

### 2.3. Preparation of the FLICA Labeling Solution

The Image-iT LIVE Green Poly Caspases Detection Kit (Cat. No. I35104, Invitrogen, USA) and the Image-iT LIVE Green Caspase-8 Detection Kit (Cat. No. I35105, Invitrogen) were used to detect activated caspases in identifiable reticulospinal neurons of larval sea lampreys after a complete SC transection. This kit contains 1 vial (component A of the kit) of the lyophilized FLICA reagent (FAM-VAD-FMK for the detection of all activated caspases and FAM-LETD-FMK for the specific detection of activated caspase-8). The reagent associates a fluoromethyl ketone (FMK) moiety, which reacts covalently with a cysteine, with a caspase-specific aminoacid sequence (valine-alanine-aspartic acid (VAD) for the poly caspases reagent and leucine-glutamic acid-threonine-aspartic acid (LETD) for the caspase-8 reagent). A carboxyfluorescein group (FAM) is attached as a fluorescent reporter. The FLICA reagent interacts with the enzyme active center of an activated caspase via the recognition sequence, and then attaches covalently through the FMK moiety [22].

To prepare the 150x FLICA reagent stock solution, 50 *μ*L of DMSO (component D of the kit) was added to the vial containing the lyophilized FLICA reagent and mixed until the reagent was completely dissolved. The unused portion of the 150x FLICA reagent can be stored in small aliquots protected from light at −20°C, and the reagent will be stable for several months.

### 2.4. Detection of Active Caspases in Whole-Mounted Brain Preparations

Brains from control noninjured animals and animals that survived for two weeks posttransection were dissected out and stripped of choroid plexus. Fresh dissected brains were immediately incubated in 150 *μ*L of phosphate buffered saline (PBS) containing 1 *μ*L of the 150x FLICA labeling solution at 37°C for 1 hour. Then, the brains were washed 9 × 15 min at room temperature (protected from light) on a nutator using 1x wash buffer. The 1x wash buffer was prepared from 10x apoptosis wash buffer (component F of the kit) by adding 9 parts of deionized water to 1 part of 10x apoptosis wash buffer. After washes, the posterior and cerebrotectal commissures of the brain were cut along the dorsal midline, and the alar plates were deflected laterally and pinned flat to a small strip of Sylgard (Dow Corning Co., USA). Brains were fixed in 4% paraformaldehyde in PBS overnight at 4°C. Next, the brains were washed 4 × 15 min with PBS, mounted on Superfrost Plus glass slides (Fisher Scientific, MA, USA), and coverslipped using Prolong (Invitrogen) as an antifade reagent.

### 2.5. Controls

In all experiments, brains of noninjured animals were processed in parallel with brains of experimental animals. Photomicrographs were taken using a 10x objective and under the same conditions of exposure time, and camera/microscope settings for those animals processed in parallel.

 As a control for the specificity of labeling, the brains of 2 SC transected animals were first incubated in PBS containing the pan caspase inhibitor Z-VAD-FMK (Promega, USA) at a concentration of 40 *μ*M for 1 hour at 37°C. Following this treatment, brains were processed for poly caspase FLICA labeling (see above).

### 2.6. Imaging and Preparation of Figures

Photomicrographs were taken using a Nikon Eclipse 80i microscope equipped with a CoolSNAP ES (Roper Scientific, USA) camera. Brightness and contrast were minimally adjusted using Adobe Photoshop CS4 software and lettering was added. Schematic drawings were carried out using CS BioDraw Ultra software.

## 3. Results

### 3.1. Pattern of Neuronal Labeling with FLICA

**Figure 2:**
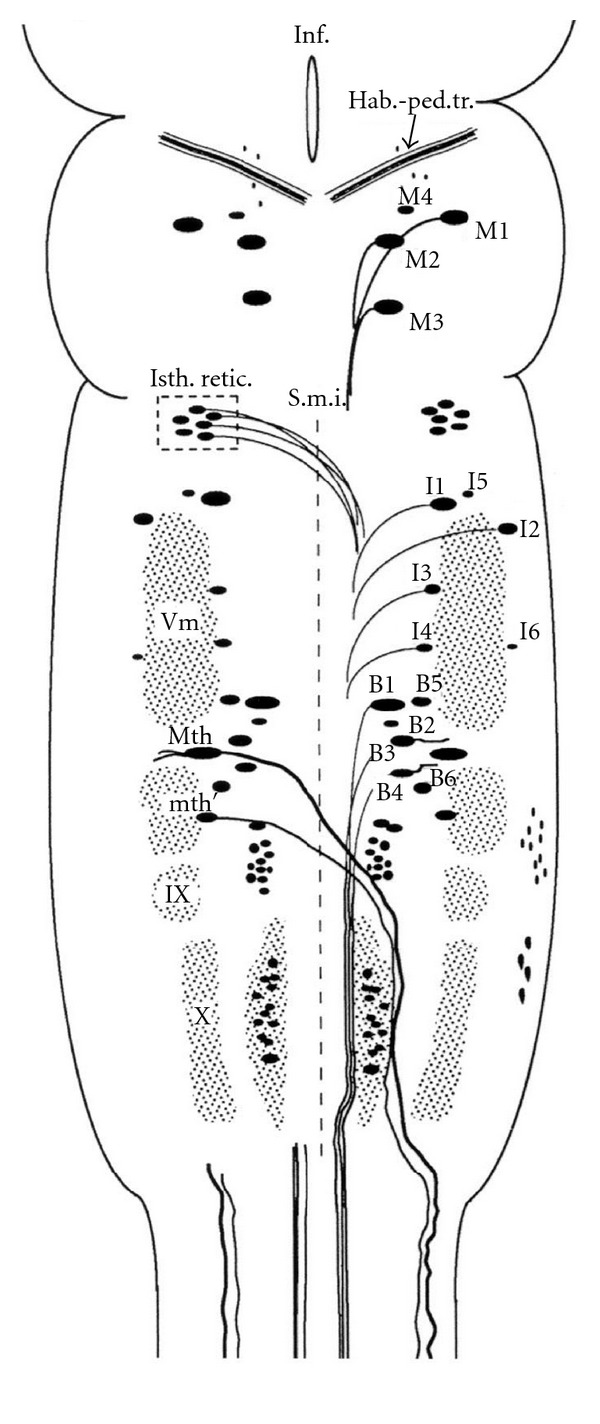
Schematic drawing of a dorsal view of the sea lamprey brain showing the location of neuronal groups and identifiable reticulospinal neurons including giant Müller cells, a pair of Mauthner neurons (Mth), and a pair of auxiliary Mauthner neurons (mth'). Three pairs of Müller cells are identified in the caudal diencephalon (M1, M2, and M4) and one pair in the mesencephalon (M3). In the rhombencephalon, two pairs of Müller cells are identified in the anterior rhombencephalic reticular nucleus of the isthmic region (I1 and I2) and four in the middle rhombencephalic reticular nucleus or bulbar region (B1–4). Recent studies have identified additional large neurons: the I3–I6 and the B5 and B6 neurons. For abbreviations, see list. (Reproduced from [21]).

**Figure 3:**
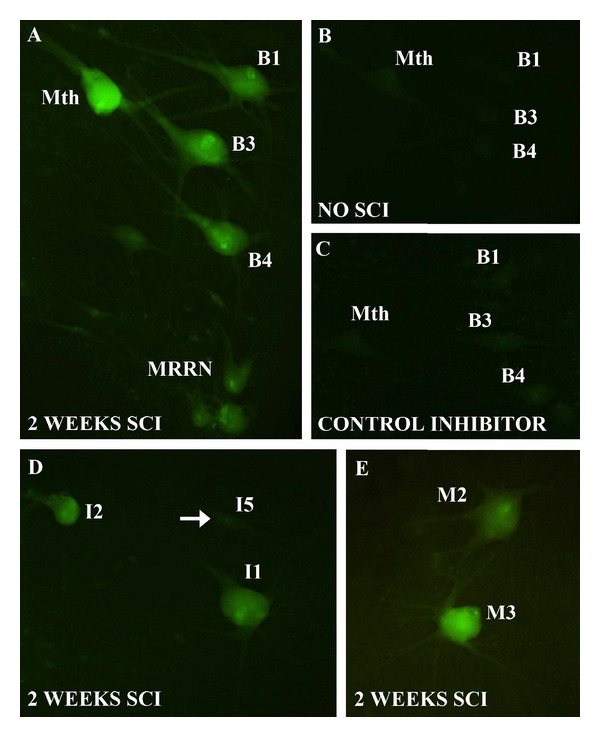
Photomicrographs of dorsal views of whole-mounted brains of larval sea lampreys show activated caspases in identified reticulospinal neurons after a complete SC transection as revealed by FAM-VAD-FMK labeling (green channel). (a) Photomicrograph of the rhombencephalon of a larval sea lamprey shows FAM-VAD-FMK labeling in the Mth, B1, B3, and B4 neurons 2 weeks after a complete SC transection. Note also the presence of labeled small-unidentified neurons of the MRRN. (b) Photomicrograph of the rhombencephalon of a larval sea lamprey shows the absence of FAM-VAD-FMK labeling in the Mth or bulbar neurons of noninjured animals. (c) Photomicrograph of the rhombencephalon of a larval sea lamprey shows the absence of FAM-VAD-FMK labeling after a complete SC transection in brains incubated with Z-VAD-FMK prior to the incubation with the FLICA reagent. (d) Photomicrograph of the rostral rhombencephalon of a larval sea lamprey shows the presence of FAM-VAD-FMK labeling in the I1 and I2 neurons 2 weeks after a complete SC transection. The arrow points to a nonlabeled I5 cell. (e) Photomicrograph of the mesencephalon/diencephalon of a larval sea lamprey shows FAM-VAD-FMK labeling in the M3 and M2 neurons 2 weeks after a complete SC transection. Rostral is up in all figures. The ventricle is at the right in all figures. For abbreviations, see list.

Whole-mounted brain preparations preserve three-dimensional information, which allows the rapid and accurate identification of labeled neurons. The lamprey brainstem can be studied in whole-mounted preparations because of its flat shape and because the lack of myelin [23] makes the CNS translucent. A schematic map of spinal projecting neurons of the sea lamprey brain is shown in Figure 2. These 36 large identified reticulospinal neurons include giant Müller cells and the Mauthner neurons and have a complex architecture (Figure 2). Apoptotic death of spinal-projecting neurons was induced by axotomy after a complete SC transection (see above). Incubation of the brains with the poly caspase FLICA reagent (FAM-VAD-FMK) revealed activated caspases in identified reticulospinal neurons of the brainstem two weeks after the SC transection (Figure 3(a)) but not in neurons of control animals without SC transection (Figure 3(b)). Intense FAM-VAD-FMK labeling was mainly observed in the soma of identifiable reticulospinal neurons known to be “bad regenerators” and “poor survivors” (the M1, M2, M3, I1, I2, Mth, B1, B3, and B4 neurons; Figures 3(a), 3(d), and 3(e)) and in smaller unidentified neurons of the middle (Figure 3(a)) and posterior (not shown) rhombencephalic reticular nuclei.

**Figure 4:**
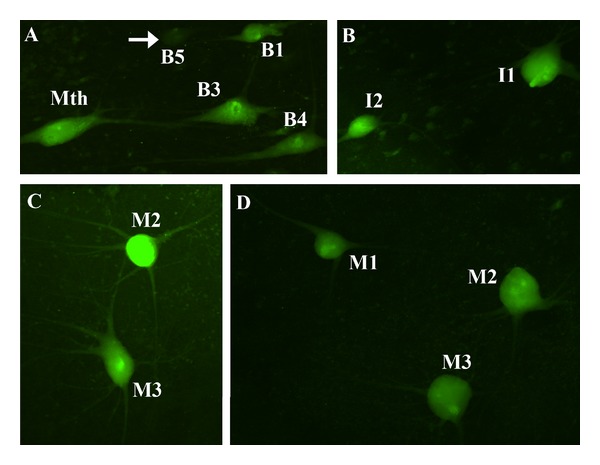
Photomicrographs of dorsal views of whole-mounted brains of larval sea lampreys show activated caspases in identified reticulospinal neurons after a complete SC transection as revealed by FAM-LETD-FMK labeling (green channel). (a) Photomicrograph of the rhombencephalon of a larval sea lamprey shows FAM-LETD-FMK labeling in the Mth, B1, B3, and B4 neurons 2 weeks after a complete SC transection. Note also a nonlabeled B5 cell (arrow). (b) Photomicrograph of the rostral rhombencephalon of a larval sea lamprey shows FAM-LETD-FMK labeling in the I1 and I2 neurons 2 weeks after a complete SC transection. (c) Photomicrograph of the mesencephalon/diencephalon of a larval sea lamprey shows FAM-LETD-FMK labeling in the M3 and M2 neurons 2 weeks after a complete SC transection. (d) Photomicrograph of the mesencephalon/diencephalon of a larval sea lamprey shows FAM-LETD-FMK labeling in the M1, M2, and M3 neurons 2 weeks after a complete SC transection. Rostral is up in all figures. The ventricle is at the right in all figures. For abbreviations, see list.

FAM-VAD-FMK FLICA reagent detects any active caspase. It is well known that alternative stimuli could activate different caspases. For example, the extrinsic or death receptor pathway activates the initiator caspase-8, whereas the intrinsic or mitochondrial pathway activates the initiator caspase-9. Therefore, in separate experiments we used a FLICA kit for the specific detection of caspase-8 to determine if we could detect specific active caspases in a wholemount brain preparation. The caspase-8 specific FLICA reagent, FAM-LETD-FMK, was the probe in these experiments. Neuronal apoptosis was again induced by axotomy due to a complete SC transection (see above). In animals studied two weeks after the SC transection, intense FAM-LETD-FMK labeling appeared in the same identifiable reticulospinal neurons as when we used the poly caspase FLICA reagent (the M1, M2, M3, I1, I2, Mth, B1, B3, and B4 neurons; Figure 4) and in smaller unidentified neurons of the middle and posterior rhombencephalic reticular nuclei (not shown). As in the case of the poly-caspase FLICA reagent, FAM-LETD-FMK labeling did not occur in normal animals without SC transection (not shown).

### 3.2. Pretreatment of Cells with Unlabeled Pan-Caspase Inhibitor as a Control of Specificity

The specificity of FLICA labeling was assessed in control experiments by preincubation of the lamprey whole-mounted brains with the pan-caspase competitive inhibitor Z-VAD-FMK. Prior exposure to this unlabeled inhibitor of caspases totally prevented subsequent labeling with the poly caspase FLICA reagent: specific poly caspase FLICA labeling did not appear in the brains of experimental animals two weeks after a complete SC transection (Figure 3(c)), thus supporting specificity of the FLICA reaction.

## 4. Discussion

Caspase activation is a hallmark of apoptosis and FLICA reagents have been previously used to detect activated caspases in cell culture [13]. Exposure of live cells to FLICA results in the rapid uptake of these reagents followed by their covalent binding to active caspase enzymes in apoptotic cells (Figure 1). Unbound FLICA reagent is removed from the nonapoptotic cells by rinsing with wash buffer (Figure 1; [13]). Since the cells have to be alive when they are incubated with the FLICA reagent, this method was only previously used to detect activated caspases in cell culture assays [13]. Here, we reported a method based on FLICA reagents to detect neuronal apoptosis *ex vivo* in whole-mounted brain preparations of lampreys. We modified the standard manufacture's FLICA protocol by adding additional washes after incubation with the FLICA reagent and extending the time of each wash. We made these modifications because our experiments used whole-mounted brains rather than cells in culture. Importantly, we showed that, because FLICA labeling is not lost after paraformaldehyde fixation, it should also be possible to combine FLICA labeling with the subsequent detection of other apoptotic or molecular markers by immunohistochemistry or by *in situ *hybridization. This method for detecting activated caspases in the lamprey brain offers advantages over methods that use commercially available antibodies generated against caspase aminoacidic sequences of mammals, which may lack specificity and/or antibody penetration in whole-mounted lamprey brain preparations.

The larval sea lamprey is an extremely useful model for studying retrograde neuronal death after axotomy. In lampreys, identifiable reticulospinal neurons with a low regenerative ability die after a complete SC transection [19]. The appearance of TUNEL staining in these identifiable neurons after axotomy has previously indicated that the death is apoptotic [19]. Two weeks after a complete SC transection, activated caspases were detected only in identified reticulospinal neurons (M1, M2, M3, I1, I2, B1, B3, B4, and Mth) that have a low probability of regeneration [24] and survival [19, 20] after axotomy as shown by Nissl, TUNEL [19, 20], or Fluoro-Jade C [20] straining at later time points. Our present results support the idea that axotomy activates a process of apoptotic cell death in these neurons. There are two main apoptotic pathways, the extrinsic or death receptor pathway and the intrinsic or mitochondrial pathway, with different caspases involved in the initiation or promotion of each pathway (see [11]). Determining the specific apoptotic pathway that is activated after axotomy will be critical for developing therapies to protect neurons from dying and promote regeneration. Our results show not only that FLICA reagents can be used to detect specific activated caspases in the lamprey whole-mounted brain but also that activated caspase-8 appears in spinal-projecting neurons 2 weeks after axotomy. An important implication of this observation is that the extrinsic or death receptor pathway of apoptosis is likely to be activated in these neurons (see above). These results in the lamprey brain are in agreement with previous reports of caspase-8 retrograde activation in retinal ganglion cells after transection of the optic nerve in rats [25, 26] and in olfactory receptor neurons after bulbectomy in mice [27].

## 5. Conclusions

We have developed a novel method for detecting activated caspases in a nonmammalian vertebrate brain that does not rely upon availability of specific antibodies. Application of this new methodology to whole-mounted brain preparations offers great opportunities for increasing our understanding of the molecular mechanisms responsible for apoptosis initiation in lampreys and, more generally, in the many additional nonmammalian vertebrates and invertebrates for which specific antiactivated caspase antibodies are not available.

## Abbreviations

B1–B6: Müller cells of the bulbar region (middle rhombencephalic reticular nucleus)
hab.-ped. tr.: Habenulopeduncular tract
I1-I6: Müller cells of the isthmic region
IX: Glossopharyngeal motor nucleus
inf.: Infundibulum
isth. retic.: Isthmic reticular formation
M1–M4: Müller cells 1 to 4
MRRN: Middle rhombencephalic reticular nucleus
Mth: Mauthner cell
mth': Auxiliary Mauthner cell
SCI: spinal cord injury
s.m.i.: Sulcus medianus inferior
Vm: Trigeminal motor nucleus
X: Vagal motor nucleus.
